# Editorial: Women in invertebrate physiology: 2021

**DOI:** 10.3389/fphys.2023.1146392

**Published:** 2023-01-31

**Authors:** Sylvia Anton

**Affiliations:** Institute for Genetics, Environment and Plant Protection, INRAE, Institut Agro, University Rennes, CEDEX, Angers, France

**Keywords:** insects, nematodes, annelids, neurobiology, stress physiology, aging

Physiology and biological sciences in general, are still dominated by contributions from male researchers worldwide, with less than 30% of scientists being women. The Women in Invertebrate Physiology Research Topic is part of a series of Frontiers Research Topics highlighting contributions of female scientists to the respective fields. These showcases are meant to encourage female scientists to pursue scientific careers in spite of often unfavorable working conditions for women, especially with young children at key moments of career decisions. The Research Topic also intends to support the battle against gender discrimination, which has still a long way to go within the scientific world.

In this Research Topic of five original research articles, with female first-and/or lead authors, various aspects of invertebrate physiology in different model organisms are covered and illustrate the creativity and innovation achieved by female researchers. Three articles present different approaches to insect physiology and two articles investigate the role of specific genes in the physiology of annelids and nematodes. Uebi et al. describe the neural mechanisms involved in the aversive effects of cuticular hydrocarbons of a native Japanese ant species on invasive ant species. Interestingly a specific nest-mate recognition compound of the native species causes indiscriminate activation within the primary olfactory center of the invasive species, creating neural disarray. The authors discuss the importance of their findings for the development of control measures for invasive ant species. Damulewicz et al. report important influences of light exposure during development on sleep regulation and underlying physiological mechanisms in adult *Drosophila melanogaster*. A lack of light exposure in larvae affected the expression of genes coding for neurotransmitters and neuropeptides, as well as for their receptors and reduced the size of the mushroom bodies significantly. The authors show furthermore the importance of chryptochrome-dependent photoreception in the brain for the observed behavioral and physiological effects. Physiological mechanisms involved in the resistance of mosquito larvae to the neonicotinoid insecticide acetamiprid were investigated by Samal et al. Using a laboratory selection approach with acetamiprid-exposed yellow fever mosquito larvae, the authors evaluate levels of detoxification enzymes and mutations in an acetylcholinesterase gene in susceptible and resistant strains. Their results show that a combination of target site mutation and metabolic detoxification cause acetamiprid resistance.

De la Fuente and Novo present an *in silico* analysis of small heat shock proteins, i.e., proteins with a critical role in stress physiology throughout the animal kingdom, in annelid worms, a taxon including species with widely varying life styles and habitats. This study identifies and characterizes for the first time this type of protein in annelids and reveals a large diversity throughout the phylum. Both monomeric and dimeric α-crystallin domains were frequently found. Physicochemical analysis indicates that monomeric small heat shock proteins cluster in two different subgroups, one close to the same type of proteins in metazoans, the other closer to proteins in plants and bacteria. Thus annelids provide interesting models to further understand the evolution and highly divergent functions of small heat shock proteins. Samaro et al. investigated the mechanisms underlying the inability to enter dauer diapause under environmental stress of *unc-33* mutants of the nematode *Caenorhabditis elegans*. Dauer diapause is characterized by long survival without feeding and uncoordinated locomotion. The *unc-33* gene is among others involved in axonal outgrowth through microtubule orientation in neuronal processes. The authors show that the dauer defects in *unc-33* mutants can be rescued by the introduction of the *daf-7(e1372)* mutation, but uncoordinated locomotion in dauers is not restored. This indicates that the DAF-7 pathway, regulating growth and development and allowing dauer arrest in *C. elegans*, might be defect in *unc-33* mutants, but that DAF-7 seems not to impact regulation of axonal outgrowth in motor neurons.

